# Generation of a highly efficient and tissue-specific tryptophan hydroxylase 1 knockout mouse model

**DOI:** 10.1038/s41598-018-36097-6

**Published:** 2018-12-05

**Authors:** Hyeongseok Kim, Yeong Gi Kim, Wonsuk Choi, Joon Ho Moon, Inseon Hwang, Kyuho Kim, Vijay K. Yadav, Gerard Karsenty, Ji-Seon Jeong, Hail Kim

**Affiliations:** 1 Graduate School of Medical Science and Engineering, Korea Advanced Institute of Science and Technology, Daejeon, 34141 Republic of Korea; 2 Biomedical Science and Engineering Interdisciplinary Program, Korea Advanced Institute of Science and Technology, Daejeon, 34141 Republic of Korea; 3 Department of Genetics and Development, Columbia University Medical Center, New York, NY 10032 USA; 4 Center for Bioanalysis, Division of Chemical and Medical Metrology, Korea Research Institute of Standards and Science, Daejeon, 34113 Republic of Korea

## Abstract

Recent studies on tissue-autonomous serotonin (5-hydroxytryptamine [5-HT]) function have identified new roles for 5-HT in peripheral organs. Most of these studies were performed by crossing mice carrying the *Tph1*^*tm1Kry*^ allele with tissue specific Cre mice. In the present study, we found that 5-HT production was not completely abolished in *Tph1*^*tm1Kry*^ KO mice. The residual 5-HT production in *Tph1*^*tm1Kry*^ KO mice is attributed to the expression of a truncated form of TPH1 containing the catalytic domain. Hence, in an effort to obtain mice with a *Tph1* null phenotype, we generated mice harboring a new *Tph1* floxed allele, *Tph1*^*tm1c*^, targeting exons 5 and 6 which encode the catalytic domain of TPH1. By crossing the new *Tph1* floxed mice with villin-Cre or insulin-Cre mice, we observed near-complete ablation of 5-HT production in the intestine and β cells. In conclusion, this improved *Tph1* floxed mouse model will serve as useful and accurate tool for analyzing peripheral 5-HT system.

## Introduction

Serotonin (5-hydroxytryptamine [5-HT]) is a monoamine neurotransmitter synthesized from the essential amino acid tryptophan. The biosynthesis of 5-HT is regulated by tryptophan hydroxylase (TPH), which is the rate-limiting enzyme for 5-HT synthesis. Since the discovery of two isotypes of TPH (TPH1 and TPH2), which display distinct tissue expression patterns^1^, it has become well accepted that central and peripheral 5-HT systems are functionally separated. This spatial separation is attributable to the inability of 5-HT to cross the blood-brain barrier; thus, 5-HT is biosynthesized centrally by TPH2 and peripherally by TPH1. 5-HT has diverse functions in both neuronal and peripheral tissues. Not only is neuronal 5-HT known to modulate sleep, mood and appetite, which are under the control of the central nervous system^2–5^, but is also known to regulate gastrointestinal motility, which is controlled by the enteric nervous system^6^. In contrast to the well-known functions of neuronal 5-HT, the roles of 5-HT in peripheral tissues are less clear. Peripheral 5-HT has been shown to regulate liver regeneration, bone formation, immune responses, and energy metabolism^7–10^. More than 90% of 5-HT in the body is peripheral 5-HT, the majority of which is produced by enterochromaffin cells of the gut mucosa and stored in platelets. Recent studies have shown that pancreatic β cells and adipocytes also produce 5-HT, catalyzed by TPH1^11,12^.

Several *Tph1* knockout (KO) alleles have been generated in mice to study the function of 5-HT in peripheral tissues. The first *Tph1* KO mouse demonstrated the duality of the 5-HT system, which is spatially separated into neuronal and peripheral compartments^1^. This mouse lacked the capacity for liver regeneration^7^, and its insulin secretion was impaired^13^. Studies in another whole body *Tph1* KO mouse revealed important roles of peripheral 5-HT in cardiac function and fetal development^14–17^. Yadav and coworkers generated the first conditional *Tph1* KO allele, which led to the discovery of previously unappreciated functions of 5-HT in bone regulation and energy metabolism^8,12,18,19^. Although the initial *Tph1* floxed allele proved useful for studying functions of peripheral 5-HT in different tissues, the resulting *Tph1* KO mice still contained 5-HT positive cells in the gut mucosa, indicating residual 5-HT synthesis and thus incomplete abolishment of TPH1 activity in the gut. All currently available KO mice have been generated by targeting exons 2 and/or 3 instead of exons 5 and 6, the latter of which encode the catalytic domain of TPH1. Therefore, all previously generated *Tph1* KO alleles may potentially express aberrantly spliced *Tph1* transcripts containing exons 5 and 6, producing proteins with TPH1 catalytic activity despite deletion of exon 2 and/or 3 of *Tph1*.

In this study, we generated a new conditional *Tph1* allele with Cre-mediated KO potential by targeted insertion of loxP sites flanking exons 5 and 6 encoding the catalytic domain of TPH1 protein. By crossing the new *Tph1* floxed mice with tissue-specific Cre mice, we demonstrate highly efficient reduction in 5-HT synthesis in peripheral tissues. These mice will be valuable for studying the function of 5-HT in peripheral tissues, including the intestine and pancreatic β cells.

## Results

### 5-HT production in tissue-specific *Tph1* KO mice

**Figure 1:**
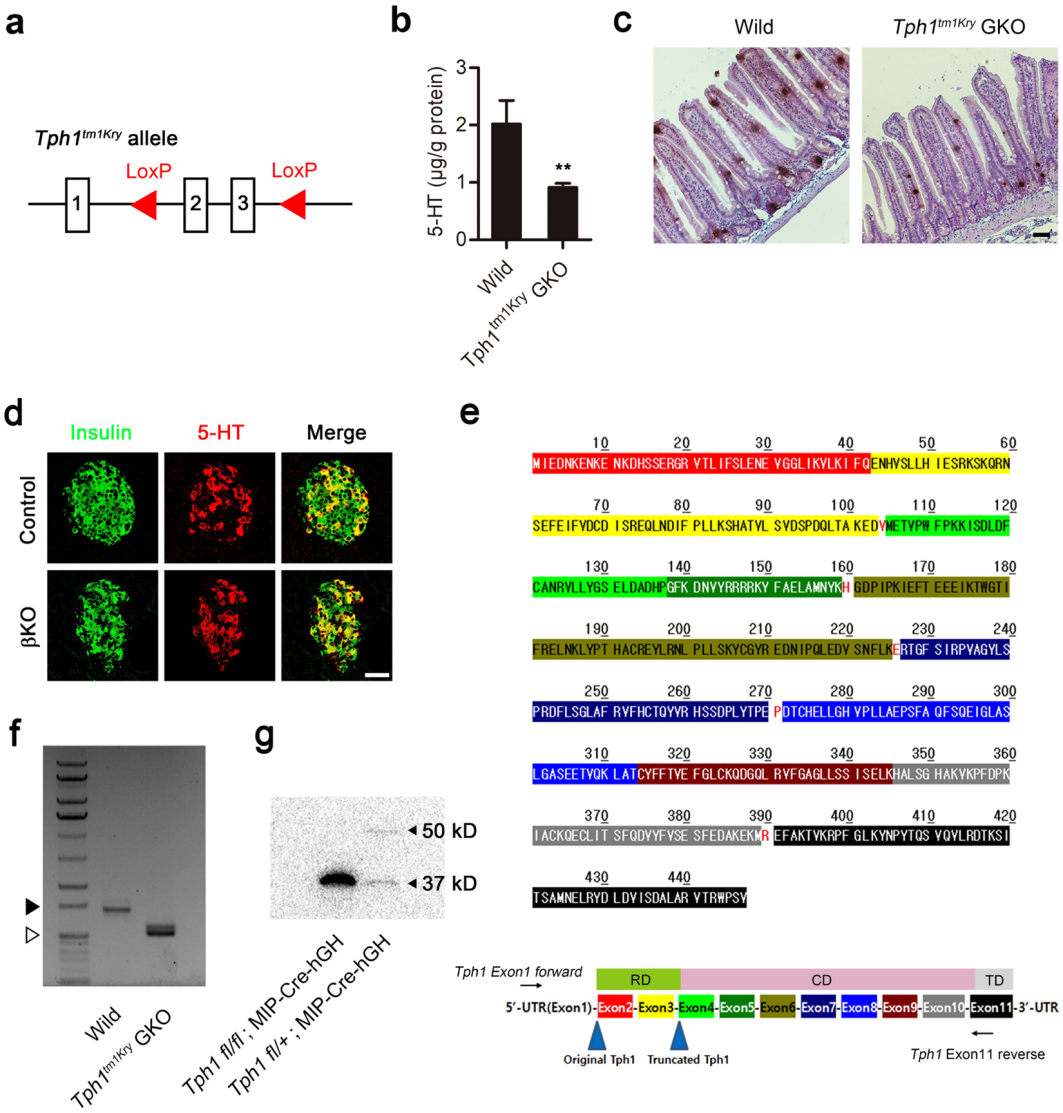
*Tph1* KO mice constructed using *Tph1*^*tm1Kry*^ retains partial TPH activity. (**a**) Genomic structure of *Tph1*^*tm1Kry*^. (**b**) Concentration of jejunal 5-HT in wild type and *Tph1*^*tm1Kry*^ GKO mice (*n = *3–7 per group). All data are presented as means ± standard error (**P < 0.01 vs. wild type, Student’s *t*-test). (**c**) Representative immunohistochemical staining of 5-HT in the jejunum of wild type and *Tph1*^*tm1Kry*^ GKO mice. Scale bar, 50 μm. (**d**) Representative immunofluorescence staining of pancreatic islets in *Tph1*^*tm1Kry*^ βKO mice (n = 3 per group). Pancreatic sections were stained for insulin (green) and 5-HT (red). Control: MIP-Cre-hGH; βKO: *Tph1*^*tm1Kry*^ βKO. Scale bar, 50 μm. (**e**) The amino acid sequence of TPH1. Red letters indicate overlapping splice sites; RD, regulatory domain; CD, catalytic domain; TD, tetramerization domain. (**f**) *Tph1* transcripts of wild type mice and KO transcripts of *Tph1*^*tm1Kry*^ GKO mice in the duodenum were amplified by RT-PCR using the primers, *Tph1* Exon1 forward and *Tph1* Exon11 reverse. Empty triangles indicate 1000 bp, and black triangles indicate 1500 bp. The full-length gel is presented in Supplementary Figure S3. (**g**) TPH1 (50 kD) and truncated TPH1 (37 kD) were detected by immunoblot analyses of pancreatic islets from *Tph1*^*tm1Kry*^ βKO and hetero βKO mice. The full-length blot is presented in Supplementary Figure S2.

To date, *Tph1*^*tm1Kry*^ has been the only conditional allele available for generating tissue-specific *Tph1* KO mice using the Cre-loxP system^8^. The KO strategy used to generate *Tph1*^*tm1Kry*^ involves targeting exons 2 and 3 of the *Tph1* gene (Fig. 1a). Although *Tph1*^*tm1Kry*^ KO mice have proven useful in delineating the functions of 5-HT and associated phenotypes in the gut and adipose tissues^8,12,20^, we noticed that 5-HT production was not completely abolished in the gut of gut-specific *Tph1*^*tm1Kry*^ KO (*Tph1*^*tm1Kry*^ GKO) mice. Overall, 5-HT levels were reduced by 54.7%, and 5-HT positive cells were readily detected in the jejunum of *Tph1*^*tm1Kry*^ GKO mice (Fig. 1b,c). As we previously reported, 5-HT was readily detected in pancreatic β cells of MIP-Cre-hGH mice by immunofluorescence staining (Fig. 1d) owing to increased *Tph1* expression^11,21^. To evaluate the KO efficiency of the *Tph1*^*tm1Kry*^ allele in pancreatic β cells, we generated β cell–specific *Tph1*^*tm1Kry*^ KO (*Tph1*^*tm1Kry*^ βKO) mice by crossing *Tph1*^*tm1Kry*^ mice with MIP-Cre-hGH mice. Immunofluorescence staining showed robust 5-HT production in the β cells of MIP-Cre-hGH mice; notably, this production was not reduced in β cells of *Tph1*^*tm1Kry*^ βKO mice (Fig. 1d). These data indicate that 5-HT production remained largely unchanged, even after presumed deletion of exons 2 and 3 of the *Tph1*^*tm1Kry*^ allele had occurred. One explanation for the residual TPH1 activity observed in *Tph1*^*tm1Kry*^ βKO and *Tph1*^*tm1Kry*^ GKO mice is incomplete deletion of the *Tph1*^*tm1Kry*^ conditional allele. However, given the high recombination efficiency of Villin-Cre and MIP-Cre-hGH mice, it is more likely that the Cre-recombined *Tph1*^*tm1Kry*^ allele produces aberrant *Tph1* transcripts that encode proteins with residual TPH1 catalytic activity. A detailed examination of the *Tph1* genomic structure revealed the presence of a methionine in exon 4 that could act as a start codon to produce a truncated TPH1 protein after the deletion of exons 2 and 3 in the *Tph1*^*tm1Kry*^ allele (Fig. 1e). Reverse transcription-polymerase chain reaction (RT-PCR) and subsequent sequence analyses revealed that three splice variants of *Tph1* lacking exons 2 and 3 were expressed in the duodenum of *Tph1*^*tm1Kry*^ GKO mice (Fig. 1f). These transcripts contained the start codon in exon 4, which is predicted to produce a polypeptide with the same reading frame as wild type *Tph1* (Supplementary Figure S1). Indeed, immunoblot analyses of tissue lysates from islets of *Tph1*^*tm1Kry*^ βKO mice revealed the presence of a 37 kD truncated TPH1 protein (Fig. 1g). TPH protein is comprised of a regulatory domain, a catalytic domain, and a tetramerization domain. The truncated TPH1 contained the catalytic domain and tetramerization domain. Therefore, we conclude that the sustained 5-HT production in *Tph1*^*tm1Kry*^ GKO and *Tph1*^*tm1Kry*^ βKO mice can be attributed to the expression of truncated TPH1 protein as a result of deletion of exons 2 and 3.

### Generation of new *Tph1* floxed mice and analysis of KO efficiency

**Figure 2:**
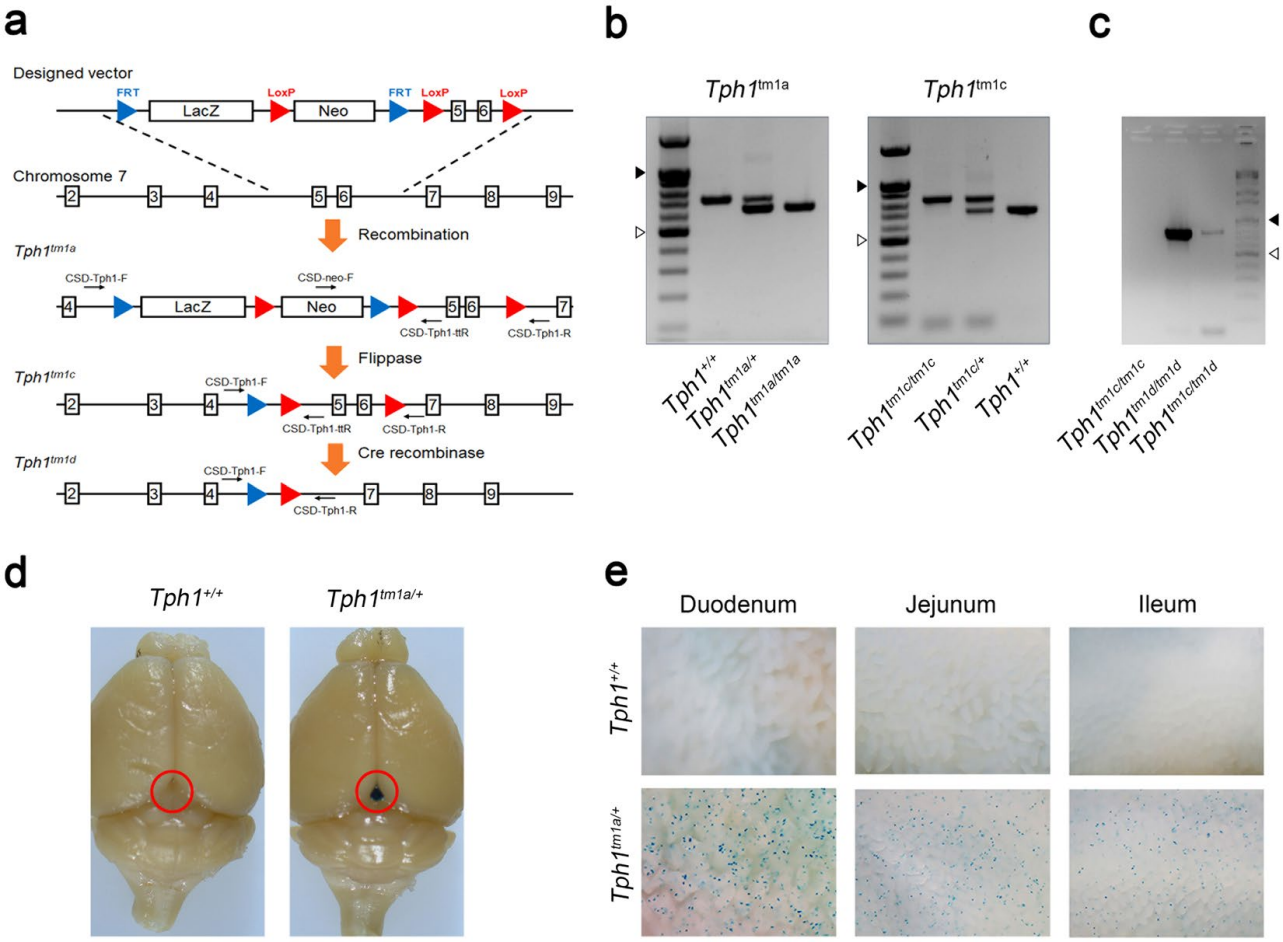
Construction of a new *Tph1* floxed mouse model. (**a**) Schematic representation of the targeting strategy for generating a new *Tph1* floxed mouse. (**b**) Genotyping of *Tph1*^*tm1a*^ and *Tph1*^*tm1c*^ mice. (**c**) Genotyping of pancreatic islets from *Tph1*^*tm1c*^ βKO mice. Empty triangles indicate 500 bp, and black triangles indicate 1000 bp. The full-length gels are presented in Supplementary Figure S3. Representative X-gal staining of the brain (**d**) and small intestine (**e**) in wild type and *Tph1*^*tm1a*^ mice (n = 3 per group). Red circles indicate the pineal grand. Images were captured using a stereomicroscope.

To completely abolish 5-HT synthesis by TPH1, we generated a new mouse harboring a *Tph1*^*tm1a*^ allele in which exons 5 and 6 encoding the catalytic domain of TPH1 were targeted. Subsequent Flp-mediated recombination of the *Tph1*^*tm1a*^ allele resulted in a *Tph1*^*tm1c*^ allele that can undergo Cre-mediated deletion of exons 5 and 6 (Fig. 2a). The *Tph1*^*tm1a*^ allele was detected by PCR amplification of genomic DNA with the primers, CSD-neo-F and CSD-Tph1-ttR, which generated a 642 bp product (Fig. 2b). Generation of the *Tph1*^*tm1c*^ allele through Flp-mediated recombination was confirmed by PCR amplification with the primers, CSD-Tph1-F and CSD-Tph1-ttR, which generated an 826 bp product (Fig. 2b). We then generated β cell–specific *Tph1*^*tm1c*^ KO (*Tph1*^*tm1c*^ βKO) mice by crossing *Tph1*^*tm1c*^ mice with MIP-Cre-hGH mice and further confirmed the deletion of exons 5 and 6 of *Tph1* in β cells of *Tph1*^*tm1c*^ βKO mice by amplifying a 793 bp genomic fragment using the PCR primers, CSD-Tph1-F and CSD-Tph1-R (Fig. 2c). The *Tph1*^*tm1a*^ allele contains a splice acceptor sequence designed to express β-galactosidase according to the endogenous pattern of *Tph1* gene expression. This β-galactosidase reporter for endogenous *Tph1* gene expression was confirmed by visualizing β-galactosidase activity in the pineal gland and gut using X-gal staining (Fig. 2d,e).

**Figure 3:**
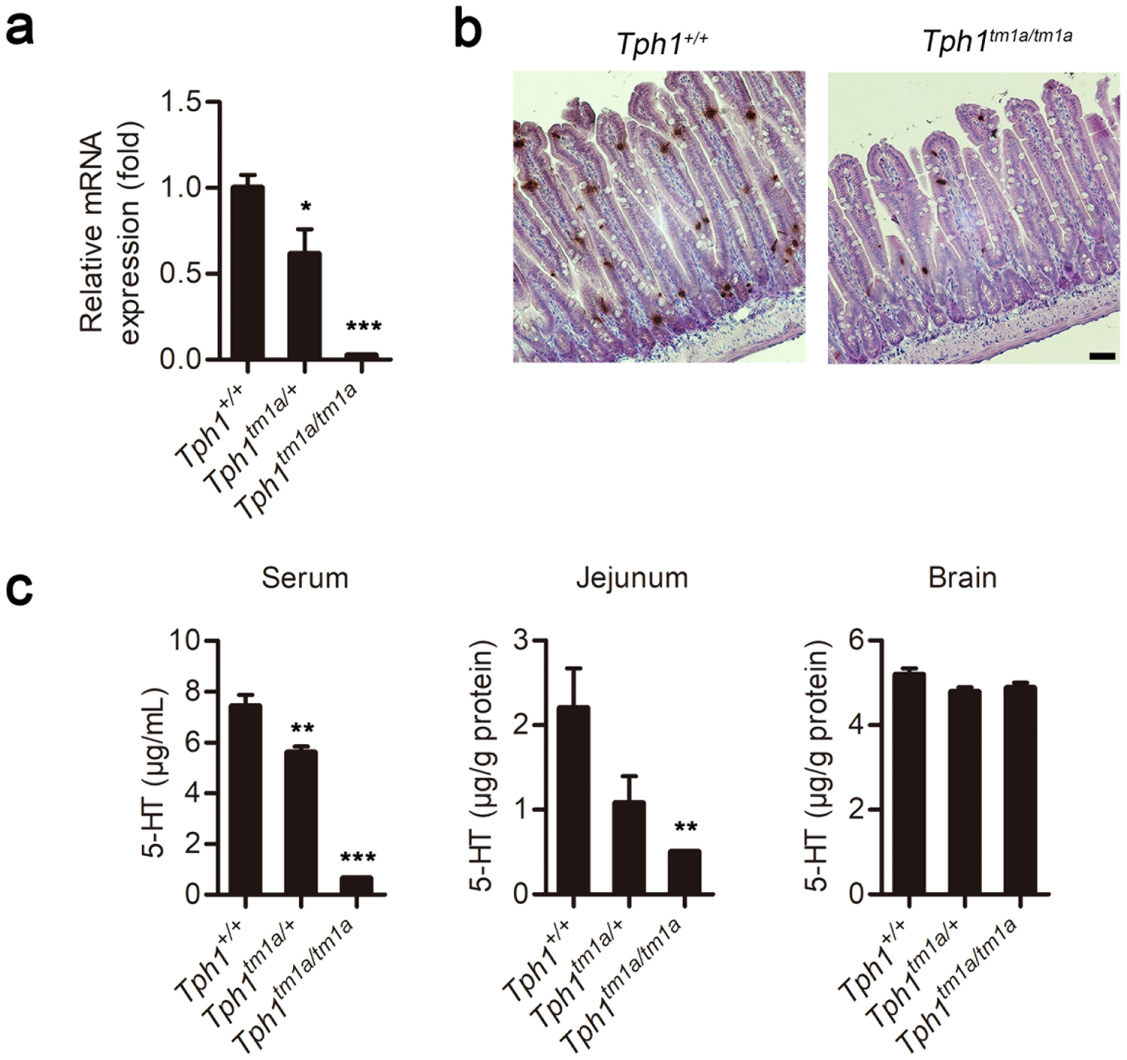
*Tph1* expression and 5-HT levels in the *Tph1*^*tm1a*^ mouse. (**a**) *Tph1* mRNA levels in the jejunum of *Tph1*^*tm1a*^ mice were determined by quantitative real time RT-PCR and expressed relative to those of wild type mice. (**b**) Representative immunohistochemical staining of 5-HT in the jejunum of wild type and *Tph1*^*tm1a*^ mice. Scale bar, 50 μm. (**c**) Levels of 5-HT in serum, the jejunum, and brain were determined by LC-MS/MS. All data are presented as means ± standard error (*n = *5–8 per group; *P < 0.05, **P < 0.01, and ***P < 0.001 vs. *Tph1*^+/+^, Student’s *t*-test).

We next evaluated the efficiency of *Tph1* KO in the newly developed *Tph1* KO mouse lines. We first examined *Tph1*^*tm1a*^ mice, which were expected to have a complete loss of the peripheral 5-HT system. Although *Tph1* mRNA levels were decreased significantly and the number of 5-HT positive cells was reduced, 5-HT positive cells were still observed in the jejunum (Fig. 3a,b). Serum 5-HT levels decreased by 91.4%, whereas jejunal 5-HT levels decreased by 77.6%. In contrast, and as expected, 5-HT levels in the brain were similar to those of wild type mice (Fig. 3c). These data indicate that ablation of 5-HT synthesis was still incomplete in the gut of *Tph1*^*tm1a*^ mice; thus, some cells in *Tph1*^*tm1a*^ mice were able to escape KO. Similar examples in which exon-trapping approaches have led to hypomorphic alleles rather than null alleles include *Slc25a21* KO mice carrying the *Slc25a21*^*tm1a(KOMP)Wtsi*^ allele^22^.

**Figure 4:**
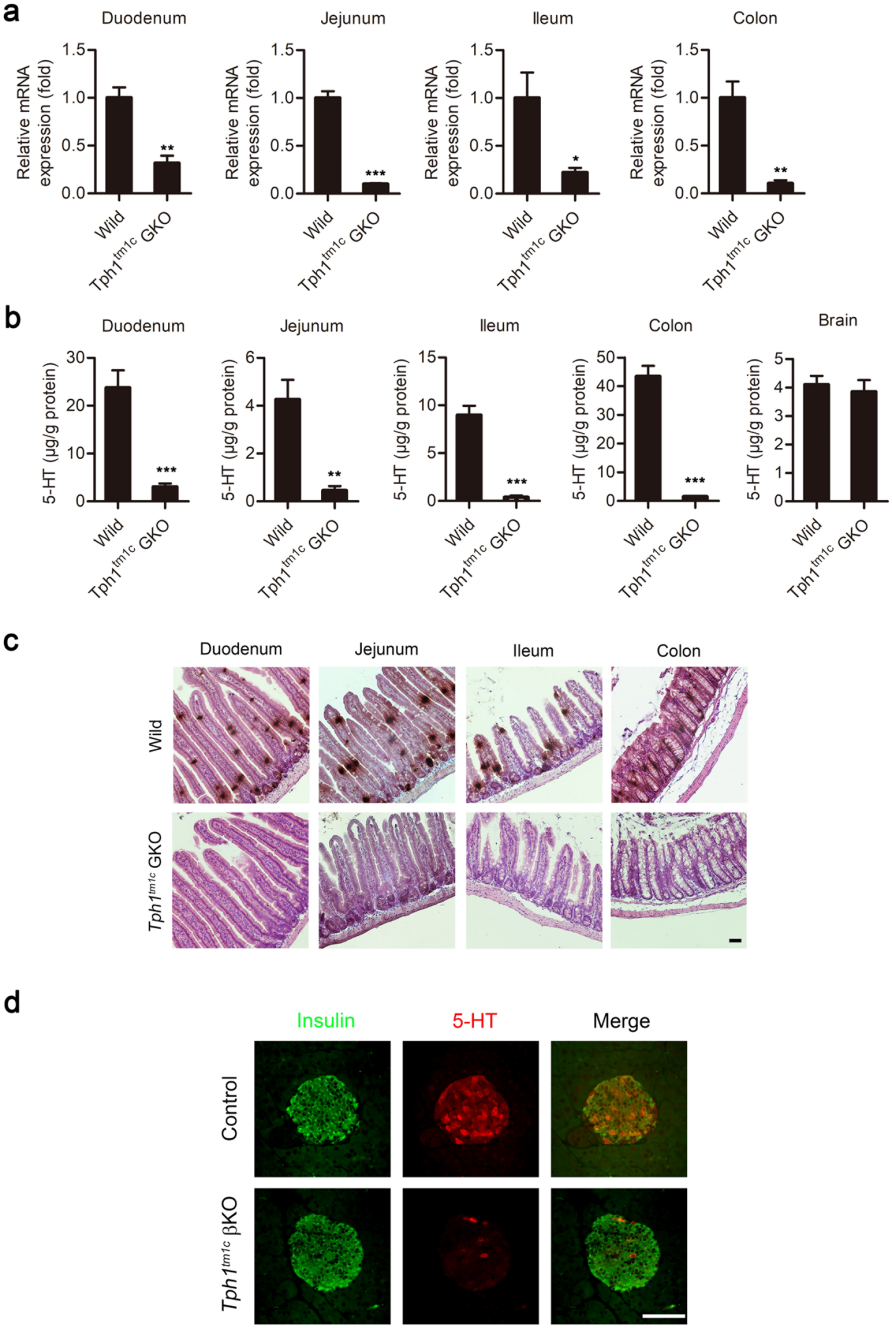
*Tph1* expression and 5-HT levels in the newly constructed *Tph1* floxed mouse model. (**a**) *Tph1* mRNA levels in the duodenum, jejunum, ileum, and colon of wild type and *Tph1*^*tm1c*^ GKO mice were determined by quantitative real time RT-PCR and are expressed relative to those of the wild type (n = 3–5 per group). (**b**) 5-HT levels in the duodenum, jejunum, ileum, colon, and brain were determined by LC-MS/MS (n = 3–8 per group). All data are presented as means ± standard error (**P < 0.01, ***P < 0.001 vs. wild type, Student’s *t*-test). (**c**) Representative immunohistochemical staining of 5-HT in the duodenum, jejunum, ileum, and colon of wild type and *Tph1*^*tm1c*^ GKO mice. Scale bar, 50 μm. (**d**) Representative immunofluorescence staining of pancreatic islets from MIP-Cre-hGH (Control) and *Tph1*^*tm1*c^ βKO mice (n = 3 per group). Pancreatic sections were stained for insulin (green) and 5-HT (red). Scale bar, 50 μm.

Next, to evaluate the tissue-specific KO efficiency, we generated *Tph1*^*tm1c*^ KO mice specific to the gut (*Tph1*^*tm1c*^ GKO) and β cells (*Tph1*^*tm1c*^ βKO) by crossing *Tph1*^*tm1c*^ mice with Villin-Cre and MIP-Cre-hGH mice, respectively. *Tph1* mRNA levels were decreased significantly in the small and large intestine of *Tph1*^*tm1c*^ GKO mice (Fig. 4a). Accordingly, tissue 5-HT levels were substantially reduced in the small and large intestines of *Tph1*^*tm1c*^ GKO mice (Fig. 4b). Considering the known recombination efficiency of Villin-Cre and activity of TPH2 in enteric nervous system, the 5-HT synthesis was nearly completely abrogated in the gut^23^. Notably, 5-HT positive cells were not observed in the gut mucosa of *Tph1*^*tm1c*^ GKO mice or β cells of *Tph1*^*tm1c*^ βKO mice (Fig. 4c,d), indicating a near-complete ablation of 5-HT production in the gut and pancreatic β cells. These data indicate that 5-HT production was completely abrogated using this newly constructed *Tph1*^*tm1c*^ mouse model.

## Discussion

5-HT plays many physiological functions in peripheral tissues as well as neuronal tissues. It has been thought that gut-derived 5-HT functions predominantly in peripheral tissues. However, recent studies have shown that cells in peripheral tissues, including pancreatic β cells and adipocytes, are capable of synthesizing and secreting 5-HT, which can play an important role in local tissues. In pancreatic β cells, 5-HT induces compensatory β cell proliferation and enhances glucose stimulated insulin secretion during pregnancy^11,24^. In adipose tissues, 5-HT functions as an obesity hormone that reduces energy expenditure and increases lipid accumulation^12,25^. As such, 5-HT can act in an autocrine or paracrine manner in peripheral tissues.

There is a limit to the reports describing the functions of peripheral 5-HT over the past decades, as most studies have been performed using conventional *Tph1* KO mice or TPH inhibitors or chemicals targeting 5-HT receptors. Therefore, it is imperative to develop efficient tools to dissect *in vivo* functions of 5-HT in local tissues and tissue-specific KO strategies offer great value as an experimental approach for assigning tissue-autonomous functions of 5-HT. To date, *Tph1*^*tm1Kry*^ is the only *Tph1* floxed allele that has been used to create tissue-specific *Tph1* KO models. *Tph1*^*tm1Kry*^ mice contributed to the initial identification of a number of peripheral functions of 5-HT. However, we found that 5-HT continues to be produced in target tissues of *Tph1*^*tm1Kry*^ KO mice, despite successful deletion of exon 2 and 3 of *Tph1*. Although *Tph1*^*tm1Kry*^ mice have proven effective in previous studies^8,12,18,20^, certain phenotypes are obscured in this setting. The amount of 5-HT required to maintain a given physiological function might differ depending on the type of tissue, phenotype to observe, or experimental conditions. Indeed, it was shown that mammary gland-specific disruption of *Tph1* did not change milk production or alveolar morphology during lactation, despite a significant decrease in 5-HT^26^. These findings, which contrast with previous reports^27,28^, may indicate that the incomplete ablation of 5-HT production affected phenotypes. Thus, a complete *Tph1* KO mouse is essential for unambiguously interpreting the full range of effects of *Tph1* deletion in specific tissues.

In an attempt to create an improved *Tph1* conditional allele, we generated *Tph1*^*tm1c*^ mice, a new *Tph1* floxed mouse model. The *Tph1*^*tm1c*^ allele was designed to result in Cre-mediated deletion of exons 5 and 6, which encode the catalytic domain of TPH1. *Tph1*^*tm1c*^ mice showed higher KO efficiency in representative 5-HT positive tissues, such as the gut and pancreatic β cells. Thus, use of the *Tph1*^*tm1c*^ mouse can be expected to reduce misinterpretations of results caused by residual 5-HT and provide more accurate analyses than were previously possible, ultimately unveiling new functions of the peripheral 5-HT system.

## Methods

### Generation of *Tph1* KO mice

The *Tph1*-targeted embryonic stem (ES) cell clone EPD0195_1_C03 was obtained from the trans-NIH Knock-Out Mouse Project (KOMP) (https://www.komp.org/). The mutant *Tph1* allele (*Tph1*^*tm1a(KOMP)Wtsi*^) contains En2 SA, FRT sites, loxP sites, lacZ, and neomycin cassettes inserted in the intron located between exons 4 and 5 of the *Tph1* gene (Fig. 2a). Targeted ES cells were injected into blastocysts of BALB/c mice, and C57BL/6N × BALB/c chimeric founders carrying the *Tph1*^*tm1a(KOMP)Wtsi*^ allele were crossed with C57BL/6J mice. Germline transmission in F1 mice was analyzed, and confirmed offspring were crossed with transgenic flippase mice to recombine the FRT sites flanking the LacZ exon trap and neomycin resistance cassette. Mice carrying the FRT-KO allele, *Tph1*^tm1c(*KOMP)Wtsi*^, were crossed with the indicated tissue-specific Cre mice. Mice carrying the *Tph1*^*tm1a(KOMP)Wtsi*^ or *Tph1*^tm1c(*KOMP)Wtsi*^ alleles were backcrossed and maintained on a C57BL/6J genetic background. For brevity, the mutant alleles are referred to in figures as tm1a (for *Tph1*^*tm1a(KOMP)Wtsi*^), tm1c (for *Tph1*^tm1c(*KOMP)Wtsi*^) and tm1d (for *Tph1*^tm1d(*KOMP)Wtsi*^), whereas mice carrying the mutant alleles *Tph1*^*tm1a(KOMP)Wtsi*^ or *Tph1*^tm1c(*KOMP)Wtsi*^ are referred to as *Tph1*^*tm1a*^ mice and *Tph1*^*tm1c*^ mice, respectively. The primers used to analyze the genotypes are listed in Supplementary Table S1.

### Animal care

*Tph1*^*tm1Kry*^ (MGI: 3837399), Villin-Cre (MGI: 2448639), and MIP-Cre-hGH^21^ mice were housed in a climate-controlled environment with specific pathogen-free (SPF) barrier facilities under a 12 h light-dark cycle, and were allowed *ad libitum* access to a normal chow diet and water. All mouse studies were approved by the Institutional Animal Care and Use Committee of the Korea Advanced Institute of Science and Technology. All experiments were performed in accordance with relevant guidelines and regulations.

### Serum and tissue preparation for measurement of 5-HT levels

Blood samples obtained by retro-orbital bleeding of mice were collected in serum-separating tubes (BD, 365967). The blood was allowed to clot for 15 min at room temperature and then was centrifuged at 2000 × g for 10 min at 4 °C. The collected serum was mixed with methanol at a 1:9 ratio, and then centrifuged at 12000 × g for 15 min at 4 °C. The resulting supernatants were immediately frozen and stored at −80 °C until further analysis.

The duodenum, jejunum, ileum, and colon were dissected and flushed with phosphate-buffered saline (PBS). The pineal gland was excised from the brain. The tissues of the duodenum, jejunum, ileum, colon, and brain were homogenized in RIPA buffer (Thermo Fisher Scientific) using a FastPrep-24 bead homogenizer (MP Biomedicals) and centrifuged at 12,000 × g for 5 min at 4 °C. The resulting supernatants were mixed with methanol at a 1:1 ratio, centrifuged at 12000 × g for 15 min at 4 °C, then immediately frozen and stored at −80 °C until further analysis.

Supernatant aliquots (100 µL) were transferred to new sample tubes and used for protein quantification by BCA assay accordance to the manufacturer’s protocol (Thermo Fischer Scientific). Separate 100-µL aliquots of supernatants were transferred to clean sample tubes and mixed with 300 µL of HPLC-grade absolute methanol (Sigma-Aldrich). After mixing, the samples were centrifuged for 15 min at 12000 × g at 4 °C. The 100 µL supernatant sample was used for measuring 5-HT by isotope-dilution liquid chromatography-mass spectrometry (LC-MS). For these assays, 20 µL of 1 mg/kg 5-HT-α,α,β,β-d^4^ Creatinine Sulfate Complex solution (CDN isotopes Inc.) was added to the sample solution, to which 100 µL of methanol was added. After mixing, the sample was evaporated under vacuum. The residue was then dissolved in 50 μL of distilled water, filtered through disposable syringe filters, and injected into the LC-MS system for analysis. The LC-MS/MS analysis was performed using an ACQUITY series UPLC system connected to a Xevo TQ-S electrospray ionization triple quadrupole-mass spectrometer (Waters, Waltham, MA, USA) at the Korea Research Institute of Standards and Science. The sample was injected into a Capcell core ADME column (2.1 mm I.D. × 100 mm, 2.6 µm; Shiseido, Japan) and eluted at a flow rate of 400 µL/min with a linear gradient from 75% mobile phase A (30 mM ammonium formate):25% mobile phase B (methanol) to 60% A:40% B for 3 min, followed by a 1-min cleaning step with 20% A:80% B and re-equilibration for 1 min. The injection volume was 3 µL, and the ion transitions (*m/z*) of 5-HT and its isotope were 159.8 > 104.9, and 163.8 > 109.1, respectively. The source ionization and fragmentation parameters were carefully optimized by monitoring the MS signal prior to analysis of the sample. The analytical software MassLynx (Version 4.1; Waters) was used for data acquisition and system control. The concentrations of 5-HT were normalized to the corresponding protein concentration (SCN).

### X-gal staining

X-Gal (5-bromo-4-chloro-3-indolyl-β-D-galactopyranoside) staining was performed as described by Seymour and coworkers^29^. The brain and small intestine were fixed by placing in ice-cold tissue fixative (4% paraformaldehyde, 2 mM MgSO_4_, 5 mM EGTA) for 1 h. Fixed tissues were washed in rinse solution A (2 mM MgCl_2_, 5 mM EGTA in buffer) for 30 min at room temperature, followed by washing in rinse solution B (2 mM MgCl_2_, 0.01% w/v sodium deoxycholate, 0.02% w/v Nonidet P-40 in buffer) for 5 min at room temperature. The tissues were then incubated overnight at 37 °C in stain base (2 mM MgCl_2_, 0.01% sodium deoxycholate, 0.02% Nonidet P-40, 5 mM K_3_Fe(CN)_6_, 5 mM K_4_Fe(CN)_6_·6H_2_O, and 1 mg/mL X-gal in 100 mM Sorensen’s phosphate buffer, pH 7.4). The stained tissues were then washed three times in PBS. Images were analyzed using a stereomicroscope (SZ61, Olympus).

### Immunohistochemistry and immunofluorescence staining

Immunostaining was performed as previously described^21^. Tissues were fixed in 10% neutral buffered formalin solution (Sigma-Aldrich) for 4 h at room temperature and washed with deionized water for 1 h at room temperature, after which they were paraffin-embedded and sliced into 4 μm-thick sections. After deparaffinizing and rehydrating, tissue sections were subjected to antigen retrieval by heating in sodium citrate buffer (10 mM sodium citrate, pH 6.0) and then blocked by incubating in 5% donkey serum for 30 min at room temperature. Thereafter, sections were incubated with guinea pig anti-insulin (1:1000; Dako, A0564) or rabbit anti-5-HT (1:1000; ImmunoStar, 20080) antibodies for 16 h at 4 °C.

Immunohistochemical analyses were performed using a VECTASTAIN ABC HRP Kit (PK-4001; Vector Labs), according to the manufacturer’s instructions. Sections incubated in primary antibody were washed with the supplied buffer and subsequently incubated in biotinylated anti-rabbit antibody for 30 min at room temperature. The tissues were then incubated with VECTASTAIN ABC Reagent for 30 min, followed by incubation in DAB peroxidase substrate solution (SK-4100; Vector Labs). The sections were subsequently counterstained and mounted.

For immunofluorescence staining, sections that had been incubated with primary antibody were washed in PBS and incubated for 2 h at room temperature with either of the following secondary antibodies: fluorescein isothiocyanate (FITC)-conjugated donkey anti-guinea pig IgG (1:1000; Jackson ImmunoResearch), rhodamine conjugated donkey anti-rabbit IgG (1:1000; Jackson ImmunoResearch), or Alexa Fluor 647 conjugated donkey anti-mouse IgG (1:1000; Jackson ImmunoResearch). Images were analyzed using a confocal microscope (LSM 780; Carl Zeiss) and a bright-field microscope (DS-Ri2; Nikon).

### Quantitative real time polymerase chain reaction (RT-qPCR)

The duodenum, jejunum, ileum, and colon were individually homogenized using FastPrep-24, and total RNA was extracted using TRIzol (Thermo Fisher Scientific). cDNA was synthesized from total RNA using High Capacity cDNA Reverse Transcription Kits (Thermo Fisher Scientific). The cDNA (10 ng/μl per reaction) was analyzed by real time PCR using a Viia7 system (Thermo Fisher Scientific) and Power SYBR Green PCR Master Mix (Thermo Fisher Scientific). The primers used for analyzing gene expression levels are provided in Supplementary Table S1.

### Immunoblot analysis

Pancreatic islets were isolated as previously described^21^ and lysed in RIPA buffer containing protease inhibitor cocktail (Sigma-Aldrich). The protein concentration was determined using the Pierce BCA Protein Assay Kit (Thermo Fisher Scientific). Proteins in whole-cell lysates were separated by sodium dodecyl sulfate-polyacrylamide gel electrophoresis (SDS-PAGE) on a 10% polyacrylamide gel and transferred to a polyvinylidene difluoride membrane. The membrane was blocked in 5% (w/v) skim milk in TBST (150 mM sodium chloride, 20 mM Tris, 0.1% Tween-20, pH 7.6) and incubated overnight at 4 °C with a rabbit anti-TPH1 antibody (1:1000; Cell Signaling, #12339). After washing three times in TBST (10 min each) at room temperature, the membrane was incubated with HRP-conjugated anti-rabbit IgG secondary antibody (1:5000; Cell Signaling, #7074) for 1 h. After washing three times in TBST (10 min each) at room temperature, immunoreactive proteins were detected using Pierce ECL Plus Western Blotting Substrate (Thermo Fisher Scientific). Images were analyzed using a ChemiDoc MP System (Bio-Rad).

### Statistical analyses

All data are presented as means ± standard error. The significance of differences between two sample means was assessed by Student’s t-test. *P*-values < 0.05 were considered statistically significant; individual p-values (*P < 0.05, **P < 0.01, and ***P < 0.001) are indicated in figures and figure legends.

## Electronic supplementary material

Supplementary information

## Data Availability

The datasets generated during and/or analyzed during the current study are available from the corresponding author on reasonable request.
