# Supplementary material for: Generation of a highly efficient and tissue-specific tryptophan hydroxylase 1 knockout mouse model
Source: Sci Rep. 2018 Dec 5;8:17642. doi: 10.1038/s41598-018-36097-6 (PMC6281741; doi:10.1038/s41598-018-36097-6)

Generation of a highly efficient and tissue-specific tryptophan hydroxylase 1 knockout mouse model

Hyeongseok Kim[1,#], Yeong Gi Kim[1,#], Wonsuk Choi[1], Joon Ho Moon[1], Inseon Hwang[2], Kyuho Kim[1], Vijay K. Yadav[3], Gerard Karsenty[3], Ji-Seon Jeong[4,*], and Hail Kim[1,2,*]

[1]Graduate School of Medical Science and Engineering, Korea Advanced Institute of Science and Technology, Daejeon 34141, Republic of Korea

[2]Biomedical Science and Engineering Interdisciplinary Program, Korea Advanced Institute of Science and Technology, Daejeon 34141, Republic of Korea

[3]Department of Genetics and Development, Columbia University Medical Center, New York, NY 10032, USA

[4]Center for Bioanalysis, Division of Chemical and Medical Metrology, Korea Research Institute of Standards and Science, Daejeon 34113, Republic of Korea

[#] These authors contributed equally to this work

* Corresponding authors:

Hail Kim, M.D., Ph.D.

Graduate School of Medical Science and Engineering, Korea Advanced Institute of Science and Technology

291 Daehak-ro, Yuseong-gu, Daejeon 34141, Republic of Korea

e-mail: hailkim@kaist.edu

Ji-Seon Jeong, Ph.D.

Korea Research Institute of Standards and Science

267 Gajeong-ro, Yuseong-gu, Daejeon 34113, Republic of Korea

e-mail: jsjeong@kriss.re.kr

Supplementary Table

Supplementary Table S1. Sequence of the primers used for genotyping and quantitative RT-PCR

Name	Sequence
Tph1 forward	TTCTGACCTGGACTTCTGCG
Tph1 reverse	GGGGTCCCCATGTTTGTAGT
36B4 forward	TCCAGGCTTTGGGCATCA
36B4 reverse	CTTTATCAGCTGCACATCACTCAGA
CSD-Tph1-F	TCAACATAGCCCTGAGCTCCTTAGC
CSD-neo-F	GGGATCTCATGCTGGAGTTCTTCG
CSD-Tph1-ttR	ATGCAAAGCTCCTACAGTGACAACG
CSD-Tph1-R	CACATTCCCTTGCAACTCTGCTACC
Tph1 exon1 forward	GACTCTTTAAACTCCAGTGG
Tph1 exon11 reverse	TCACACACTGGGCCACCTGG

Supplementary Figure

Supplementary Figure S1. Sequence of splicing variant of *Tph1* inTph1^{tm1Kry} GKO mice. (**a**) Sequence of *Tph1* transcript of wild type mice and KO transcript of *Tph1^{tm1Kry}* GKO mice. Red blocks indicate first ATG of transcript. Purple letters indicate exon 1 of *Tph1*. Red letters indicate exon 2 of *Tph1*. Yellow letters indicate exon 3 of *Tph1*. Green letters indicate exon 4 of *Tph1*.

```
Tph1                        GACTCTTTAAACTCCAGTGGCTCTGAGGTGAGTGGGTGAGTGGGATAGCCCCTCCCTGGG
Tph1tm1Kry GKO variant 1    GACTCTTTAAACTCCAGTGGCTCTGAGGTGAGTGGGTGAGTGGGATAGCCCCTCCCTGGG
Tph1tm1Kry GKO variant 2    GACTCTTTAAACTCCAGTGGCTCTGAGGTGAGTGG-------------------------
Tph1tm1Kry GKO variant 3    GACTCTTTAAACTCCAGTGGCTCTGAG---------------------------------

Tph1                        ACATCGGATCAGAAGACTCCCAGCAAGGACGGGATCAACTTCTAGTAGGAACCAGATTCA
Tph1tm1Kry GKO variant 1    ACATCGGATCAGAAGACTCCCAGCAAGGACGGGATCAACTTCTAGTAGGAACCAG-----
Tph1tm1Kry GKO variant 2    ------------------------------------------------------------
Tph1tm1Kry GKO variant 3    ------------------------------------------------------------

                              Start codon
Tph1                        CCATGATTGAAGACAACAAGGAGAACAAAGAGAACAAAGACCATTCCTCCGAAAGAGGGA
Tph1tm1Kry GKO variant 1    ------------------------------------------------------------
Tph1tm1Kry GKO variant 2    ------------------------------------------------------------
Tph1tm1Kry GKO variant 3    ------------------------------------------------------------

Tph1
Tph1tm1Kry GKO variant 1    ------------------------------------------------------------
Tph1tm1Kry GKO variant 2    ------------------------------------------------------------
Tph1tm1Kry GKO variant 3    ------------------------------------------------------------

Tph1                        TCTTCCAGGAGAATCATGTGAGCCTGTTACACATCGAGTCCCGGAAATCAAAGCAAAGAA
Tph1tm1Kry GKO variant 1    ------------------------------------------------------------
Tph1tm1Kry GKO variant 2    ------------------------------------------------------------
Tph1tm1Kry GKO variant 3    ------------------------------------------------------------

Tph1                        ATTCAGAATTTGAGATATTTGTTGACTGCGACATCAGCCGAGAACAGTTGAATGACATCT
Tph1tm1Kry GKO variant 1    ------------------------------------------------------------
Tph1tm1Kry GKO variant 2    ------------------------------------------------------------
Tph1tm1Kry GKO variant 3    ------------------------------------------------------------

Tph1                        TCCCCCTGCTGAAGTCGCACGCCACCGTCCTCTCGGTGGACTCGCCCGATCAGCTCACTG
Tph1tm1Kry GKO variant 1    ------------------------------------------------------------
Tph1tm1Kry GKO variant 2    ------------------------------------------------------------
Tph1tm1Kry GKO variant 3    ------------------------------------------------------------

Tph1                        CGAAGGAAGACGTTATGGAGACTGTCCCTTGGTTTCCAAAGAAGATTTCTGACCTGGACT
Tph1tm1Kry GKO variant 1    ------------TTATGGAGACTGTCCCTTGGTTTCCAAAGAAGATTTCTGACCTGGACT
Tph1tm1Kry GKO variant 2    ------------TTATGGAGACTGTCCCTTGGTTTCCAAAGAAGATTTCTGACCTGGACT
Tph1tm1Kry GKO variant 3    ------------TTATGGAGACTGTCCCTTGGTTTCCAAAGAAGATTTCTGACCTGGACT
```

Supplementary Figure S2. Images of the original western blot in Figure 1g. (**a**) Full scanned chemiluminescence image. (**b**) Image of membrane with molecular weight markers. (**c**) Image merged with protein bands and molecular weight markers.

a

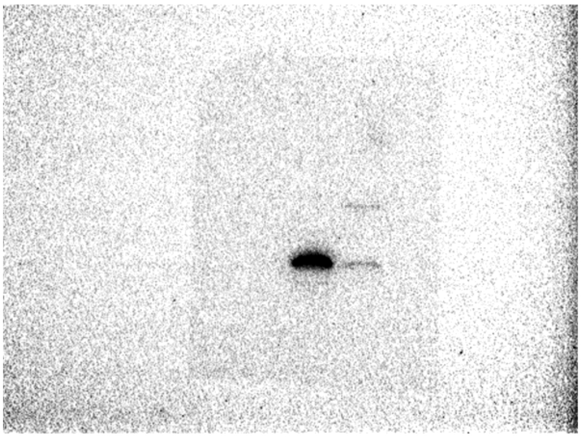

b

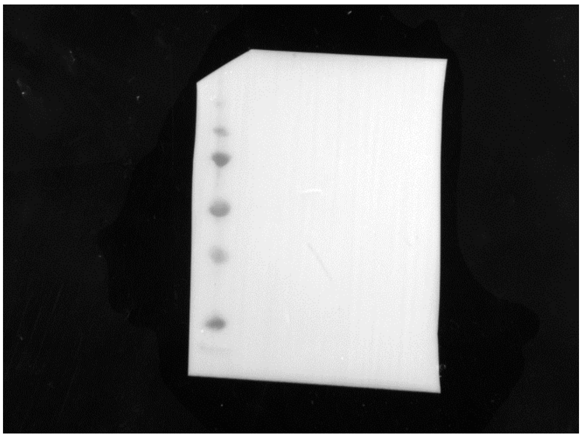

c

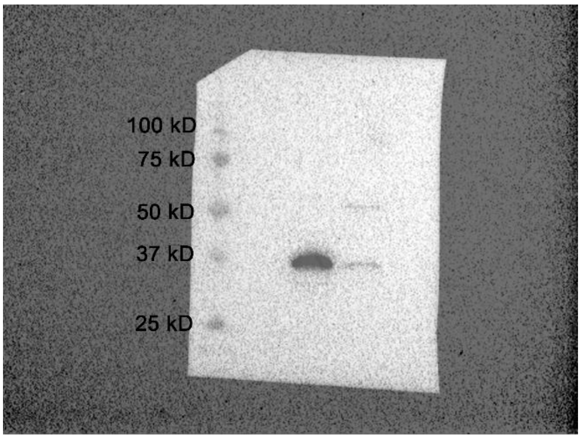

Supplementary Figure S3. Images of original gels in Figure 1 and 2. Full scanned image of Figure 1f (**a**), left panel of Figure 2b (**b**), right panel of Figure 2b (**c**), and Figure 2c (**d**).

a

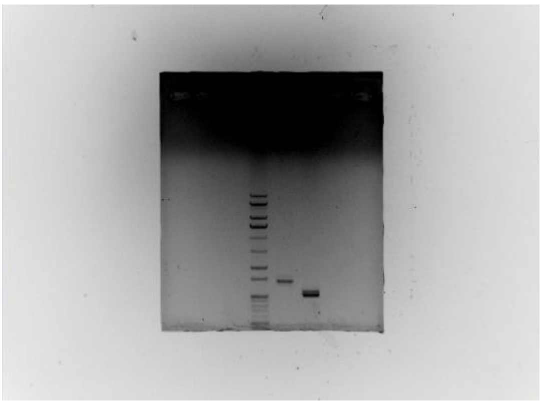

b

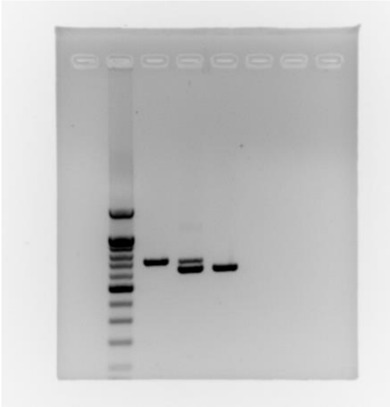

c

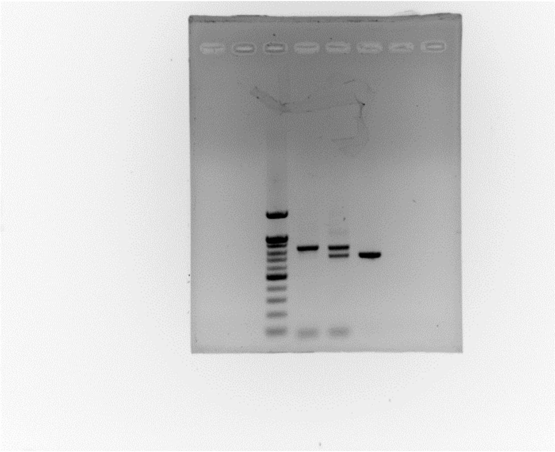

d

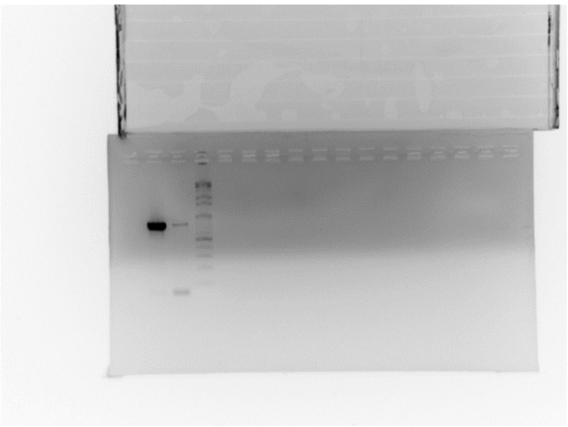

Supplement: Supplementary file 1 — Supplementary information [file 41598_2018_36097_MOESM1_ESM.pdf]
